# Personality Traits and Inflammation in Depressive Disorders

**DOI:** 10.3390/jcm11071974

**Published:** 2022-04-01

**Authors:** Katarzyna Wachowska, Piotr Gałecki, Janusz Szemraj, Janusz Śmigielski, Agata Orzechowska

**Affiliations:** 1 Department of Adult Psychiatry, Medical University of Lodz, 90-419 Lodz, Poland; katarzyna.wachowska@umed.lodz.pl (K.W.); piotr.galecki@umed.lodz.pl (P.G.); 2 Department of Medical Biochemistry, Medical University of Lodz, 90-419 Lodz, Poland; janusz.szemraj@umed.lodz.pl; 3 Department of Health Sciences, Academy of Applied Sciences in Konin, 62-510 Konin, Poland; janusz.smigielski.stat@gmail.com

**Keywords:** depression, inflammation, personality traits, neuroticism

## Abstract

Depression is a psychiatric disorder of heterogeneous etiology. One of the leading theories suggests an inflammatory background to it. It is often found in the scientific literature that certain personality traits, such as high neuroticism, low extroversion and conscientiousness, are being associated with depression. We combined biochemical tests of IL-1 and IL-6 serum levels and scores in the personality test EPQ-R among 50 depressed patients and 37 healthy participants. The results confirmed increased serum levels of IL-1 and IL-6 in a study group when compared to healthy volunteers. Additionally, personality traits (psychoticism and neuroticism) were increased in the depressed group when compared to healthy volunteers. The authors analyzed correlations in both groups. However, only one statistically significant link was observed in IL-6 and K levels (scale associated with the need for social acceptance) in the control group.

## 1. Introduction

Depression is one of the most common psychiatric disorders. According to WHO estimates, it affects over 300 million people worldwide, which is equivalent to 4.4% of the world’s population [1]. The etiology of this complex, heterogeneous and multifaceted disorder is not yet known. One of the top contemporary theories is associating mood disorder with systemic inflammation [2,3]. It has been well proved by increased levels of inflammatory (IL-1, Il-6, Crp TNFα and many others) markers documented in scientific research [4,5,6,7,8,9,10,11]. However, the origins of inflammation have not yet been clearly found [12]. Some research suggests that certain personality traits, such as neuroticism and psychoticism, might also be associated with depressive symptoms [13] or even predict them [14]. Alizadeh et al. (2018) in a big study observed that high level of neuroticism is strongly associated with depression, stress and anxiety, whereas other personality traits are largely protective in terms of mental health [13]. A meta-analysis of World research on associations between personality and mental health problems (such as depression, dysthymia, social and specific phobias and substance abuse) suggested that all the aforementioned disorders were associated with high scores on neuroticism scale and low on conscientiousness, in many studies also with low extroversion [15]. The neuroinflammatory theory of depression proposes an explanation of the observed phenomenon [16]. In essence, it states that early stages of development of both brain and immune system and their various interactions are crucial in creating predisposition to further mental disorders [16,17].

The aim of the presented research was to examine several personality traits (extroversion/introversion, neuroticism, psychoticism) and two examples of inflammatory markers–IL-1 and IL-6 among patients suffering from depressive disorders and comparing those parameters with scores achieved by healthy volunteers. Our basic assumption was that personality traits commonly associated with depression–neuroticism will be higher among depressed patients and that it will correlate with increased levels of inflammatory markers. However, it would be difficult to determine which factor is causal in that regard. We decided to use EPQ-R as one of the relatively rarely used questionnaires (most often used are NEO-FFI-R and MMPI) to make our approach more novel.

## 2. Materials and Methods

The study involved 87 participants, male and female. Written, informed consent was obtained from all participants prior to the procedure. The study design was constructed coherently, under the principles of the Declaration of Helsinki, and gained approval of the Bioethics Committee at the Medical University of Lodz, consent number RNN/45/15/KE of 17/03/2015.

The group consisted of two subgroups: 50 were diagnosed with recurrent depressive disorders, while 37 were healthy control subjects. A summary of the study group and the control group characteristics is presented in Table 1. In Table 2, a comparison between the groups with respect to gender is presented.

All participants were Poles coming from central Poland. Participants were included in the study on the basis of diagnosis of depressive disorders according to ICD-10 and DSM 5 classifications. Patients with co-morbid substance abuse (apart from tobacco), severe head trauma in past medical history and dementia were excluded from the study.

Severity of depression assessment. The Hamilton Rating Depression Scale (HDRS) in version 17 was used to objectify the severity of depressive symptoms at the time of the study. The scale was filled in by the researcher (psychologist) during an interview with each patient.

Personality assessment was performed with EPQ-R questionnaire. It consisted of 100 yes/no questions. Participants filled in the test by themselves. The results are presented in four main scale categories. Three of them—Psychoticism (P), Extraversion (E) and Neuroticism (N)—relate to the basic dimensions of personality. The so-called control scale—Lies (K)—allows one to assess the tendency to present oneself in a better light.

Biochemical methods. A sample of blood (10 mL) was taken from participants who gave informed consent for blood tests (50 in depressed group, 30 in healthy control group).

Determination of protein expression.

Determining protein concentration.

An amount of 150 µL of the reaction mixture was added to tubes containing 150 µL of serum, diluted 10 times in 10 mM of phosphate-buffered saline, pH 7.4, and incubated (2 h, 37 °C). In order to specify protein concentration, an analytical curve for serum albumin was determined. Both the examined samples and the reference samples were prepared in parallel in three repetitions. Sample absorbance was measured using Multiskan Ascent Microplate Photometer (Thermo Labsystems, Gulph Mills, PA, USA) at λ = 562 nm, and total protein concentration was calculated from the standard curve equation.

Enzyme-linked immunosorbent assay (ELISA).

The concentration of proteins IL1β and IL6 in the serum of the patients was determined using Human IL1β and IL6 Elisa Kit (R D Systems, Minneapolis, MN, USA) according to the protocols provided by the manufacturer. β-actin was used for endogenous control of protein concentration in the samples and determined with the help of Human Actin Beta (ACTb) ELISA Kit (BMASSAY) based on the manufacturer’s recommendations. An amount of 100 μL of serum (ρprotein = 0.5 mg/mL) was added to tubes coated with antibodies specific to the analyzed proteins and incubated (1.5 h, 37 °C). The content was removed, and the tubes were rinsed three times in 10 mM of phosphate-buffered saline and incubated (1 h, 37 °C) with 100 μL of biotinylated antibodies specific to the analyzed proteins. Then, the content was removed, and the tubes were rinsed three times in 10 mM of phosphate-buffered saline and incubated (30 min, 37 °C) with 100 μL of ABC Working Solution. The content was removed, and the tubes were rinsed five times in 10 mM of phosphate-buffered saline and incubated (10 min, 37 °C) with 90 μL of TMB substrate. After adding 100 μL of TMB Stop Solution, the absorbance of the samples was measured using Multiskan Ascent Microplate Photometer (Thermo Labsystems, Gulph Mills, PA, USA) at λ = 450 nm. In order to determine protein concentration, analytical curves for the analyzed proteins were produced.

Statistical analysis. The data were verified for normality of distribution and equality of variances. Correlations between the levels of interleukins and scores in personality testing (EPQ-R) were analyzed with the Spearman’s rank correlation coefficient. Gender structure analysis was performed by the Chi-square test. The Mann–Whitney U-test was used to compare the average values received. Statistical analysis was performed using the Statistica 13th CSS program. The results of the quantitative variables are presented as a mean ± SD (standard deviation), median. The limit of statistical significance was set at *p* < 0.05 for all the analyses. Shapiro–Wilk test was used to verify normality. We used Dunn’s test, which reduces the risk of making a type II error.

## 3. Results

### 3.1. Inflammatory Markers

The results obtained from biochemical analysis of blood samples in the study group and the control group are presented in Table 3.

Statistical analysis of the variables was performed with the use of Mann–Whitney U-test (due to non-parametric variables). The results are presented in Table 4.

Statistically significant differences between depressed patients and healthy controls are observed in terms of the levels of IL-1, IL-6 and the expression of IL-6, but not in the expression of IL-1β. Both IL-1 and IL-6 serum levels were higher in the depressed group when compared to the control group. Alternatively, the measures of expression of IL-1β and IL-6 were higher in the control group when compared to the study group, although only statistically significant in the IL-6β expression level.

### 3.2. Personality Traits

The results obtained from the personality questionnaire in both groups are presented in Table 5.

Statistical analysis of the variables was performed with the use of Mann–Whitney U-test (due to non-parametric variables). The results are presented in Table 6. Statistically significant differences between depressed patients and healthy controls are observed in scores P, representing Psychoticism/Socialization dimension, and N, representing Neuroticism/Stability dimension. Both scores, P and N, were higher in the depressed group when compared to the control group. Additionally, the mean score in K dimension was higher in the study group, however, not reaching statistically significant level. Alternatively, the E-I dimension score was higher in the control group, although likewise not with statistically significant difference. Table 7 demonstrates that gender in the study group did not interfere with results of personality testing.

### 3.3. Correlation Coefficients among Serum Levels of Interleukins and Results of Personality Test and Depression Severity

Correlations between serum levels of interleukins (IL-1β, IL-6), levels of their expressions and results of personality test (E-I, P N, K) were analyzed with the Spearman’s rank correlation coefficient. Correlations were performed for each group separately. The results are presented in Table 8 and Table 9.

No statistically significant correlations were observed in the study group. In the control group one, a positive, statistically significant correlation between IL-6 and scale K (Lies) was obtained.

## 4. Discussion

The presented study confirmed previous observations associated with increased inflammatory markers among patients suffering from depression [4,5,6,7,8,9,10]. Serum levels of IL-1 and IL-6 were higher among depressed patients than healthy controls. Additionally, the study group and the control group differ with statistical difference in some of the measured personality traits: neuroticism and psychoticism. Both of those traits were significantly higher among depressed subjects when compared to healthy controls. However, in the presented study, those two domains (personality and inflammation) were not correlated.

Neuroticism is defined as a tendency to experience negative emotions, to be rather self-centered and to react to various stimuli in an anxious way. Many scientific reports associate it with both somatic and mental health disturbances [18,19,20]. Psychoticism is a heterogeneous construct, commonly associated with impulsivity and sensation seeking. Originally, it was also associated with liability to have a psychotic episode and aggression [21].

Many studies report associations between inflammatory markers and personality traits. Schmidt et al. (2018) presented original research associating serum levels of pro-inflammatory agents—IFN-γ, IL-5 and IL-12—with overall neuroticism. TNF-α, IFN-γ, IL-5, IL-12 and IL-13 were also related to the severity of depressive symptoms, as well as the somatic–affective and cognitive dimensions of depression. That finding suggested that pro-inflammatory IFN-γ, IL-5 and IL-12 might be identified as mediators of the positive prediction of depression severity by the degree of neuroticism [22]. Research work among the Swiss Community Sample suggested that certain personality traits might be associated with low-grade inflammation–extroversion, whereas others, such as conscientiousness, with lower-grade risks. In the cited research, there was no correlation between measured inflammatory marker–IL-6 and neuroticism and agreeableness [23]. Additionally, Polish research among depressed patients suggested the existence of links between so-called neurotic triad (measured with the use of Minnesota Multiphasic Personality Inventory (MMPI-2)) to inflammatory process (defined in that article by expression at the level of mRNA and protein of MnSOD, MPO and metalloproteinases 2 and 9) [24]. Spanish research also indicated that high neuroticism (among other risk factors) is associated with depression [25]. Additionally, Swedish research presented existing link between IL-6 and neuroticism [26]. Chen et al. (2021) presented results of a longitudinal research on early adulthood depression and associated it, among many other things, positively with neuroticism and psychoticism and negatively with extroversion [27]. Zhang et al. (2021) straightforwardly stated that there is a genetic link between neuroticism and numerous health problems, including depression [28]. Dong et al. (2020) presented research combining neuroimaging and personality scores. It revealed higher neuroticism scores were associated with higher activities in several brain areas: the posterior cingulate cortex and thalamus. It was also revealed that the highly neurotic group had higher neural stress responses in precuneus and bilateral thalamus in comparison to those who scored lower on neurotic scale [29].

The research conducted so far indicates a relationship between cigarette smoking and mental illness. Smoking cessation rates remain consistently lower in depressed smokers than in the general population, highlighting the need for theory-based models of smoking and depression [30]. The literature does not give an unambiguous answer as to the direction of this correlation. A review of reports on the matter was presented by Fluharty et al. (2017). It included 148 studies assessing the relationship between smoking and depression and/or anxiety in longitudinal studies and revealed the need for further analysis of this phenomenon [31]. Almost half of the studies analyzed showed that the baseline depression/anxiety was associated with some type of subsequent smoking behavior, while more than a third of the studies revealed evidence that cigarette smoking was associated with subsequent depression/anxiety. However, there has been little research directly supporting the two-way model of smoking and anxiety, and very few studies have reported zero results. There were no clear patterns related to gender, ethnicity, clinical status, duration of follow-up or diagnostic test. The research we present consisted of collecting an interview regarding the presence of addictions, including smoking. The interview was not extended by the number of years of smoking and the time relationship between the onset of this addiction and the onset of depression symptoms and psychiatric treatment. For this reason, the authors concluded that conducting a statistical analysis separately for smoking and non-smoking patients should be considered in further studies in the future, taking into account the harmful behavior.

Although our study confirmed that both domains, personality and inflammation, are functioning differently among depressed patients and healthy controls, it failed to confirm correlations between inflammatory markers and personality traits. However, it did confirm significant differences observed between depressed and healthy subjects in both of these domains, which corresponds with previous scientific observations. Most notably, it showed higher scores in neuroticism among depressed patients. A tendency to react with anxiety to both outside and inside stimuli seems to be a well-established observation among depressed patients. According to the neuroinflammatory theory of depression, exposure to stress at the early stages of development of the brain contributes to dysregulation of immune system and nervous system [16,32]. The authors of the aforementioned theory highlight significance of epigenetic processes in creating individual predisposition to depression [16,32]. Additionally, the system of endogenous cannabinoids and the kynurenine pathway have been proposed as a common biological basis modulating both the level of inflammation and personality [33]. To summarize, the link between maladaptive personality traits (neuroticism, a tendency toward anxious reactions, and psychoticism, a tendency toward impulsivity) and depression might be explained by changes in the nervous system caused by long-term stress. Subjects prone to development of depressive disorders have a higher tendency to react in an anxious and impulsive way to objectively indifferent stimuli. Therefore, their body systems—both the immune and the nervous—are in a state of overreaction, manifested by increased levels of inflammatory markers and symptoms of depression. In addition to the observed biological stress response, personality traits seem to play an important role. Personality is not only considered to be a factor underlying mood disorders, but it is also believed that the “reaction” of the patient’s personality to the appearance of basic symptoms of the disease determines the occurrence and the nature of non-specific secondary symptoms. The relationship between depression and personality is multi-directional and figuring out what is primary and what is secondary becomes a problem. Persons with disturbed personality traits are prone to developing depression. At the same time, long-term mental illness and experiences associated with it secondarily disturb the functioning of the personality [34]. It has also been observed that patients with depression use less favorable coping strategies [35]. To make it more difficult, susceptibility to those kinds of reactions seems to be formed at a very early stage of life—during prenatal life and early childhood. Therefore, it is very important to psychologically strengthen the mechanisms of an individual, i.e., their adaptive reactions to stress when dealing with modern-world challenges.

## 5. Conclusions

Several conclusions based on the described research might be formed:

Increased serum levels of inflammatory markers IL-1 and IL-6 are observed among patients suffering from depression when compared to healthy controls.

Depressed subjects obtained significantly higher scores in Neuroticism and Psychoticism than healthy volunteers.

Extroversion and Lie scores did not differ significantly between the study and the control group.

Although the presented data confirm previous observations of increased levels of inflammatory markers among depressed patients, as well as personality traits, such as neuroticism and psychoticism, the collected data do not allow the association of inflammatory imbalance with personality traits.

The observed differences in personality traits are not associated with changes in serum levels of the examined inflammatory markers in the study group.

However, in the healthy control group, we observed a correlation between serum level of IL-6 and one of the scores in personality test K—Lies—commonly associated with the need for social acceptance. It might suggest that among non-depressed subjects, some personality traits are associated with biochemical sign of inflammatory process.

The presented study has limitations, such as a relatively low number of participants of the study, as well as of the control group, and a lack of prospective observation of depressed patients. Additionally, there are other studies available on the correlation between depression, inflammation and personality disorders. However, we tried to give a new perspective by using quite rarely used questionnaire EPQ-R. Another limitation is differences between the groups and the fact they were not equally numerous. Therefore, the obtained results need to be treated with caution and should be confirmed in further research.

## Figures and Tables

**Table 1:** Demographic characteristics of study group and control group. Parameters associated with severity of depression.

Demographic Characteristics	Study Group	Control Group
Number of participants	50	37
Age	46.34+/−11.18	40.2+/−16.42
Number of hospitalizations	2.06+/−2.11	---------
Male/Female		28/9
	40/10	
Living in a big city/small city		30/7
	34/16	
Smokers/non-smokers	32/18	8/29

**Table 2:** Gender differences between groups.

	Study Group	Control Group	All
Female	40	28	68
%	80.00%	75.68%	
Male	10	9	19
%	20.00%	24.32%	
All	50	37	87
	Chi-kwadr.	*p*	
Chi^2^ Pearsona	0.2329512	*p* = 0.62934	

**Table 3:** Inflammatory markers in the study group, the control group and comparison between them (statistically significant results marked with red colour).

	Study Group					Control Group				
Name of a Marker	Mean	Med	Min	Max	SD	Mean	Med	Min	Max	SD
IL-1 β (pg/mL)	10.75	1.0	7.8	13.2	1.45	8.28	8.05	4.80	11.10	1.38
Expression IL1 β	2.47	0.73	0.47	88.0	12.34	5.31	0.67	0.29	69.00	16.95
Il-6 (pg/mL)	6.28	6.35	3.7	9.5	1.18	5.30	5.30	0.80	7.90	1.54
Expression IL6	1.8	0.36	0.22	36.0	7.06	4.20	0.29	0.05	32.00	10.22

**Table 4:** Inflammatory markers—comparison between groups (statistically significant results marked with red colour).

	Comparison between Groups								
Name of a Marker	N	Mean	Med	Min	Max	SD	Skewness	Mann–Whitney U-Test	*p* Value
IL-1 β (pg/mL)	80.00	9.83	9.80	4.80	13.20	1.86	−0.20	5.77	0.00
Expression IL1 β	80.00	3.53	0.70	0.29	88.00	14.21	5.09	1.89	0.06
Il-6 (pg/mL)	80.00	5.91	5.95	0.80	9.50	1.40	−0.62	2.76	0.01
Expression IL6	80.00	2.69	0.33	0.05	36.00	8.40	3.36	2.88	0.00

**Table 5:** Results of Eysenck test—study group and control group (statistically significant results marked with red colour).

	Study Group						Control Group					
Name of a Parameter	*n*	Mean	Med	Min	Max	SD	N	Mean	Med	Min	Max	SD
E-I	50	10.32	10.50	4.00	18.00	4.21	37	11.59	11.00	3.00	20.00	3.75
P	50	7.74	7.00	2.00	20.00	3.24	37	5.73	5.00	1.00	14.00	3.16
N	50	19.62	21.00	9.00	24.00	3.69	37	10.89	11.00	0.00	22.00	6.20
K	50	9.76	9.50	2.00	19.00	3.59	37	8.35	8.00	2.00	20.00	3.80

**Table 6:** Results of Eysenck test—comparison between groups (statistically significant results marked with red colour).

	Comparison between Groups								
Name of a Parameter	*n*	Mean	Med	Min	Max	SD	Skewness	Mann–Whitney U-Test	*p* Value
E-I	87	10.862	11.000	3.000	20.000	4.047	0.051	−1.32	0.187549
P	87	6.885	7.000	1.000	20.000	3.339	0.966	2.55	0.010644
N	87	15.908	18.000	0.000	24.000	6.532	−0.806	6.21	0.000000
K	87	9.161	9.000	2.000	20.000	3.726	0.230	1.87	0.061855

**Table 7:** Study group results of personality testing with regard to gender.

Mann–Whitney U-Test (with the Continuity Correction)				
Gender				
Condition v2 = 1		Study group		
	Z	*p*	*n*	*n*
			Female	Male
Years of psychiatric treatment	−0.41	0.680749723	36	9
E-I	−0.11	0.9130907	40	10
P	0.96	0.338055556	40	10
N	0.49	0.627626137	40	10
K	−0.19	0.846153689	40	10
HDRS	0.83	0.409339638	38	10

**Table 8:** Correlation coefficients among serum levels of interleukins and results of personality tests—the study group.

The Study Group	Spearman’s Rank Order Correlation MD Removed in Pairs Marked Correlation Coefficients Are Significant *p* < 0.05000			
	*n*	R	Student’s *t*-Test	*p*
IL 1 β (pg/mL) and E-I	50	0.166	1.17	0.248283
IL 1 β (pg/mL) and P	50	0.245	1.75	0.086588
IL 1β (pg/mL) and N	50	0.118	0.83	0.412440
IL 1 β (pg/mL) and K	50	−0.047	−0.33	0.744995
Expression IL1 β and E-I	50	0.109	0.76	0.449149
Expression IL1 β and P	50	0.177	1.24	0.219875
Expression IL1 β and N	50	−0.149	−1.04	0.301738
Expression IL1 β and K	50	−0.061	−0.42	0.674090
IL 6 and E-I	50	0.175	1.23	0.223221
IL 6 and P	50	0.121	0.85	0.402117
IL 6 and N	50	−0.125	−0.87	0.385958
IL 6 and K	50	−0.060	−0.42	0.678712
Expression IL6 and E-I	50	0.198	1.40	0.168309
Expression IL6 and P	50	−0.055	−0.38	0.702270
Expression IL6 and N	50	−0.056	−0.39	0.698359
Expression IL6 and K	50	0.052	0.36	0.722134

**Table 9:** Correlation coefficients among serum levels of interleukins and results of personality tests—the control group (statistically significant results marked with red colour).

The Control Group	Spearman’s Rank Order Correlation MD Removed in Pairs Marked Correlation Coefficients are Significant *p* < 0.05000			
	*n*	R	Student’s *t*-Test	*p*
IL 1 β (pg/mL) and E-I	30	−0.046	−0.242	0.810528
IL 1 β (pg/mL) and P	30	0.251	1.371	0.181209
IL 1β (pg/mL) and N	30	−0.005	−0.028	0.977551
IL 1 β (pg/mL) and K	30	−0.096	−0.512	0.612616
Expression IL1 β and E-I	30	0.351	1.981	0.057523
Expression IL1 β and P	30	0.180	0.971	0.339950
Expression IL1 β and N	30	−0.182	−0.980	0.335268
Expression IL1 β and K	30	−0.175	−0.942	0.354283
IL 6 and E-I	30	0.003	0.018	0.985922
IL 6 and P	30	−0.088	−0.466	0.644998
IL 6 and N	30	0.141	0.752	0.458318
IL 6 and K	30	0.543	3.418	0.001948
Expression IL6 and E-I	30	0.148	0.793	0.434691
Expression IL6 and P	30	0.242	1.319	0.197731
Expression IL6 and N	30	0.068	0.361	0.720626
Expression IL6 and K	30	−0.248	−1.355	0.186192
